# Floral scent of the Mediterranean fig tree: significant inter-varietal difference but strong conservation of the signal responsible for pollinator attraction

**DOI:** 10.1038/s41598-023-32450-6

**Published:** 2023-04-06

**Authors:** Li Cao, Younes Hmimsa, Salama El fatehi, Bruno Buatois, Marie-Pierre Dubois, Maïlys Le Moigne, Martine Hossaert-McKey, Yildiz Aumeeruddy-Thomas, Anne-Geneviève Bagnères, Magali Proffit

**Affiliations:** 1 CEFE, CNRS/University of Montpellier/EPHE/IRD (UMR 5175), 1919 Route de Mende, 34293 Montpellier Cedex 5, France; 2 TEDAEEP Research Team, Abdelmalek Essaadi University (FPL), B. P. 745, Poste Principale, 92004 Larache, Morocco

**Keywords:** Chemical ecology, Agroecology, Plant domestication, Plant ecology, Plant signalling

## Abstract

For thousands of years, humans have domesticated different plants by selecting for particular characters, often affecting less-known traits, including the volatile organic compounds (VOCs) emitted by these plants for defense or reproduction. The fig tree *Ficus carica* has a very wide range of varieties in the Mediterranean region and is selected for its traits affecting fruits, including pollination, but the effect of human-driven diversification on the VOCs emitted by the receptive figs to attract their pollinator (*Blastophaga psenes*) is not known. In the present study, VOCs from receptive figs of eight varieties in northern Morocco, were collected at different times within the manual pollination period and analyzed by gas chromatography-mass spectrometry. Genetic analyses using microsatellite loci were performed on the same varieties. Despite strong inter-varietal differences in the quantity and relative proportions of all VOCs, the relative proportions of the four pollinator-attractive VOCs showed limited variation among varieties. There was no significant correlation between genetic markers and chemical profiles of the different varieties. While diversification driven by humans has led to differences between varieties in VOC profiles, this paper suggests that throughout the process of domestication and varietal diversification, stabilizing selection has maintained a strong signal favoring pollinator attraction.

## Introduction

Domestication is a complex process of selecting and adapting wild plant and animal species for human use. Most of the crops of today were domesticated from their wild ancestors within the past 12,000 years^1^. Human practices, through crop domestication and selection of traits, have altered the characteristics of wild ancestors to produce crop plants generally characterized by higher yield, more favorable organoleptic properties, and other characteristics such as their capacity for storage. While these traits have been selected for, others have been lost. For instance, cultivated varieties often have lower defenses and resistance to pests than their wild relatives^2,3^. There also exist differences in defenses between cultivated varieties of some crop species, such as manioc^4^, sorghum^5^, and oca^6^. Compared to plant defense, inter-varietal comparisons of floral traits related to pollinator attraction are more limited^7,8^. Nevertheless, generalist pollinators, bees in general, appear to be able to distinguish between crop varieties of the same species and it has been suggested that floral volatile organic compounds (VOCs) play a major role in this choice^9,10^. As pollination is an essential ecosystem service to crops^11^, improving our understanding of the evolutionary effects of human-driven varietal diversification on VOCs emitted by flowers involved in plant-pollinator interactions is crucial, particularly with regard to the biodiversity loss we are currently facing.

Domestication may have affected the evolution of the composition of floral VOCs through direct selection of a particular chemical composition, through the indirect effects of selection on other floral traits or on traits of other organs such as fruits, or by genetic drift. In fact, direct selection acts on floral VOCs in many aromatic plants cultivated for the perfume industry. For example, the essential oil from the true lavender, *Lavandula angustifolia*, used for perfume contains a lower camphor content than does essential oil from the wild spike lavender, *Lavandula latifolia*, and that from the hybrid lavandin, *Lavandula x intermedia*^12,13^. On the other hand, in commercial breeding programs, since floral odor is rarely targeted, unlike characteristics such as cold and disease resistance, flower shape, or vase life, floral odor is often missing in modern varieties^14^. The cause of the lack of fragrance in these flowers is often unknown. However, there is strong evidence for the existence in some plants of pleiotropic relationships between VOCs and color pigments as they often derive from common biosynthetic precursors^15^. For instance, white flowers of some plant species emit higher levels of benzenoid/phenylpropanoid scent compounds than their colored counterparts^16^. Moreover, during the domestication of fruit crops, the selection of certain fruit traits related to a particular flavor, color, or odor, probably resulted in the addition, elimination, and/or modification of various specialized metabolic pathways, leading to gain or loss of specialized metabolites^17^. These specialized metabolites, such as glycosides or ethylene, but also particular enzymes, play roles in VOC production^18^. However, despite the fact that VOCs emitted by different varieties of the same species are generally very diverse in terms of constituents and relative proportion of floral VOCs^19,20^, there are currently no studies characterizing the effect of human-driven diversification on the emission of VOCs responsible for pollinator attraction.

The Mediterranean fig tree, *Ficus carica* (Moraceae), represents one of the most appropriate biological models to explore this question. It is one of the emblematic trees of the Mediterranean region, and was domesticated around 5500 years ago^21^. Like other *Ficus* spp., trees of *F. carica* bear figs that are enclosed inflorescences termed ‘syconia’. The species is dioecious. Male trees bear figs that produce pollen and in which the specific pollinator, the fig wasp *Blastophaga psenes* (Hymenoptera: Agaonidae), develops. Female trees bear figs in which only seeds develop, but not pollinators^22^. Attraction of female *B. psenes* by VOCs emitted by receptive figs of male and female trees has already been well described^23^. In the Mediterranean region, a wide diversity of varieties of female fig trees (several hundred) have been selected. Comparatively, the classification of male fig trees into varieties is still debated. Traits of interest for human consumption in female figs that have been selected from wild figs are mainly based on fruit characteristics, including size, color, sweetness, softness, drying capacities linked to trade and storage, but also phenology, with early to late varieties in order to obtain fruits over the longest period possible. The traits selected over the centuries are also related to the type of fruit production: in some varieties the classical mutualistic interaction with the fig wasps has been maintained to produce fruit, but other varieties have been selected to produce parthenocarpic (seedless) figs that do not require pollination for fruit production^24^. In addition, the dioecious nature of figs and their manual pollination, termed caprification, have both been long well-known by Mediterranean farmers^25^. In fig cultivation, to ensure this manual pollination, male figs are traditionally either cultivated separately from the female figs (e.g. in Turkey, for the Smyrna figs) or are collected from wild trees, as in Morocco. Then, to facilitate pollination of female figs, farmers bring mature male figs, when the pollinators are ready to emerge, to the specific female fig varieties when they are receptive, ready to be pollinated. To date, no study has examined inter-varietal variations in VOCs emitted by the receptive figs.

In Morocco, the Rif region shows the highest level of varietal diversity of female fig trees^26,27^. Farmers in this region differentiate more than a hundred fig varieties, propagated by cuttings of stems^27^. Fig orchards are polyvarietal with a mean of six varieties per orchard (Hmimsa, unpublished data). In each orchard, the set of varieties present is based on complex trait preferences, with haphazard arrangements of varieties^27^. Among these traits, farmers favor varieties with different but partially overlapping receptive phases^28^. They use manual pollination to assist reproduction of female varieties that are not parthenocarpic. Even if individual tree of *F. carica* bears figs that are on the whole at the same stage of development, some small differences among figs on the same tree exist. Thus, manual pollination starts for each variety when the farmers consider that enough figs per tree are receptive, the most experienced ones referring to a change in odor as a signal for them to search for male Figs^28^, and takes place several times for each tree of each variety. However, the variation in VOC emission from receptive figs along this manual pollination period is not known.

**Figure 1:**
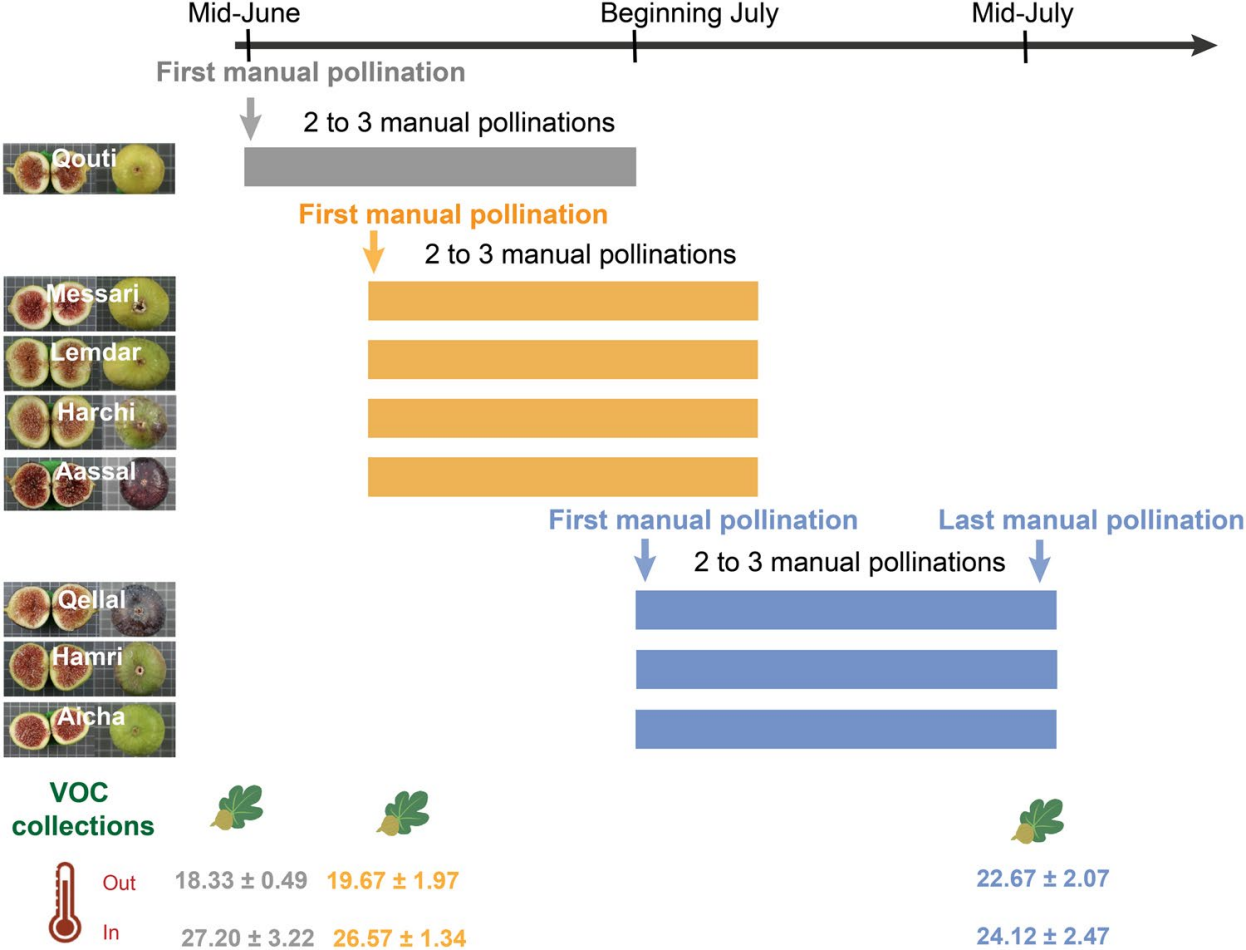
Diagram showing the timing of manual pollination and of collection of volatile organic compounds (VOCs) from receptive figs for the eight varieties of *Ficus carica* studied. The time of the year is indicated by the black arrow at the top of the diagram. For each variety, the first manual pollination time is indicated and each individual tree was pollinated two to three more times over a period of two to three weeks. Varieties with the same color (grey: Beginning; yellow: Middle; blue: End) experienced the first manual pollination at the same time. Three VOC collection sections of the same six individual trees per variety were made (indicated by the drawing of the figs). For each of these sections, the mean (± standard errors) per individual tree of the temperatures inside (in) and outside (out) the bag containing the figs are presented in degrees Celsius.

In the present study, we investigated the VOCs of receptive figs of eight non-parthenocarpic fig varieties in the Rif region in northern Morocco, in order to determine if their emission varied qualitatively but also quantitatively, particularly those responsible for pollinator attraction. Previous studies have shown that only four of the VOCs emitted by receptive figs (i.e. *S*-linalool, benzyl alcohol, (*Z*)-linalool-oxide (furanoid), and (*E*)-linalool-oxide (furanoid)), within a narrow range of proportions, are responsible for attraction of the specific fig wasp pollinator and that even a very small change in their proportions disrupts pollinator attraction^23^. Based on these results, we hypothesized that the proportions of the VOCs responsible for pollinator attraction should be highly conserved among the different varieties at receptivity, in contrast to the VOCs that play no role in pollinator attraction, as they are not affected by such stabilizing selection. As farmers decide to pollinate trees of different varieties at different times during the fig receptive season, we also hypothesized that the emission of the four attractive VOCs will show different patterns of variation between varieties during the manual pollination period, with each variety having a peak emission at the time it is pollinated. In order to test our hypothesis, we compared the VOCs collected from receptive figs of three (the first year) and six (the second year) individual trees belonging to each of eight varieties during two successive years. These varieties were among the most frequently planted in the region. In parallel, we performed a genetic comparison of the different varieties using 15 microsatellite loci to determine genetic distances. In addition, during the second year we collected and analyzed the VOCs of receptive figs of the same six trees per variety during the whole period of manual pollination at three different times during the manual pollination period: at the beginning, middle and end of this period (Fig. 1). We addressed the following questions: (1) Are there differences among varieties in the VOC composition of figs at receptive stage? (2) Is there a correlation between genetic and chemical distances in these eight varieties? (3) Over the entire manual pollination period of the figs, is there qualitative and quantitative variation in VOCs emitted by receptive figs? (4) Is the time of manual pollination of each variety associated with the peak emission of VOCs attractive to pollinators?

## Results

In total, we detected 46 VOCs and identified 35 emitted by the receptive figs of the eight varieties and during the two successive years. Receptive figs emitted floral scents mainly composed of four different chemical classes: monoterpenes, sesquiterpenes, fatty acid derivatives and benzenoids (Table 1). The blend of compounds was always dominated by terpene compounds (> 90%) in all varieties. The varieties Qouti, Lemdar and Messari released high amounts of monoterpenes (> 80%), whereas varieties Aassal, Aicha, Hamri and Qellal emitted higher amounts of sesquiterpenes (> 40%), and the variety Harchi was intermediate (monoterpenes > 60%, sesquiterpenes > 30%) (Table 1, Supplementary Fig. S1). In all varieties, the form of linalool detected was exclusively *S*-linalool. The temperature outside and inside the bag showed limited variation in both years (Fig. 1) and in all analyses, bag temperature had no significant effect on the estimated quantity or relative proportions (*p* > 0.05 for all tests).

**Table 1:** Relative proportions of all volatile organic compounds (VOCs) emitted by receptive figs of the eight varieties of *Ficus carica*.

	RI	Qouti	Messari	Lemdar	Harchi	Aassal	Qellal	Hamri	Aicha
		*N* = 9	*N* = 8	*N* = 9	*N* = 9	*N* = 9	*N* = 9	*N* = 9	*N* = 9
Benzenoids									
Benzaldehyde^a^	964	3.66 ± 3.07	0.57 ± 0.83	0	0.11 ± 0.33	0.01 ± 0.02	0.5 ± 0.84	0.12 ± 0.17	1.6 ± 1.75
Benzyl alcohol* ^a^	1036	1.41 ± 1.15	2.51 ± 2.43	4.79 ± 3.9	0.79 ± 1.12	3.28 ± 2.68	0.88 ± 0.99	0.34 ± 0.71	1.13 ± 1.54
Total percent		**5.07**	**3.08**	**4.79**	**0.9**	**3.29**	**1.38**	**0.46**	**2.73**
Fatty acid derivatives									
(*Z*)-Hex-3-enol	865	0.1 ± 0.14	0.41 ± 0.51	0.07 ± 0.12	0.08 ± 0.1	0.57 ± 1.25	1.23 ± 2.19	0	0.44 ± 0.9
(*Z*)-Hex-3-enyl acetate^a^	1006	0.02 ± 0.03	0.08 ± 0.11	0.02 ± 0.02	0.03 ± 0.05	0.06 ± 0.09	0.16 ± 0.3	0	0.45 ± 1.2
Total percent		**0.12**	**0.5**	**0.09**	**0.11**	**0.63**	**1.39**	**0.00**	**0.89**
Monoterpenes									
* α*-Pinene^a^	933	0.01 ± 0.01	0.01 ± 0.03	0.01 ± 0.01	0.05 ± 0.04	0.02 ± 0.03	0.17 ± 0.34	0.1 ± 0.11	0.1 ± 0.1
Sabinene	986	0.06 ± 0.06	0.02 ± 0.03	0.03 ± 0.06	0.15 ± 0.16	0.05 ± 0.12	0.08 ± 0.1	0.18 ± 0.21	0.01 ± 0.04
Myrcene^a^	993	0.72 ± 1.55	0.78 ± 0.94	0.32 ± 0.21	0.48 ± 0.37	0.28 ± 0.35	0.92 ± 1.42	0.02 ± 0.04	0.73 ± 1.62
Limonene^**a**^	1030	1.2 ± 1.48	0.77 ± 0.74	0.49 ± 0.42	1.57 ± 1.44	1.13 ± 0.59	3.12 ± 2.98	3.14 ± 3.7	3.18 ± 1.56
(*Z*)-*β*-Ocimene^**a**^	1039	1.14 ± 0.93	1.34 ± 1.16	1.31 ± 1.43	0.96 ± 0.62	1.49 ± 2.11	1.45 ± 1.33	0.18 ± 0.23	1.24 ± 1.19
(*E*)-*β*-Ocimene ^a^	1049	0.52 ± 1.33	0.47 ± 0.7	0.13 ± 0.08	0.11 ± 0.11	0.21 ± 0.31	0.62 ± 1.01	0.32 ± 0.25	1.06 ± 1.29
* γ*-Terpinen^a^	1061	0.12 ± 0.14	0.04 ± 0.06	0.01 ± 0.02	0.2 ± 0.14	0.21 ± 0.12	0.25 ± 0.48	0.12 ± 0.12	0.16 ± 0.15
(*Z*)-Linalool oxide (Fur.)*^a^	1074	9.94 ± 4.58	4.34 ± 2.81	5.26 ± 5.32	7.27 ± 5.48	6.03 ± 6.81	6.87 ± 6.08	2.04 ± 3.72	7.19 ± 6.2
(*E*)-Linalool oxide (Fur.)*^a^	1090	0.2 ± 0.13	0.22 ± 0.16	0.27 ± 0.16	0.16 ± 0.13	0.15 ± 0.15	0.73 ± 0.65	0.05 ± 0.07	0.26 ± 0.22
*S*-Linalool*^a^	1100	69.26 ± 18.97	81.08 ± 10.99	81.44 ± 10.93	55.71 ± 27.16	23.54 ± 24.18	36.81 ± 24.46	9.67 ± 7.31	30.21 ± 25.05
* allo*-Ocimene^a^	1131	0.41 ± 0.39	0.52 ± 0.5	0.5 ± 0.33	0.48 ± 0.5	0.38 ± 0.42	0.39 ± 0.32	0.05 ± 0.09	0.34 ± 0.53
* neo*-*allo*-Ocimene	1144	0.05 ± 0.13	0.02 ± 0.04	0.01 ± 0.01	0.02 ± 0.03	0	0.01 ± 0.02	0	0.01 ± 0.04
(*Z*)-Linalool oxide (Pyr.)	1173	0.87 ± 0.85	0.4 ± 0.23	0.32 ± 0.26	0.99 ± 0.78	0.62 ± 0.57	0.72 ± 0.7	0.15 ± 0.19	0.41 ± 0.21
(*E*)-Linalool oxide (Pyr.)	1177	0.16 ± 0.19	0.11 ± 0.08	0.04 ± 0.02	0.1 ± 0.06	0.11 ± 0.09	0.33 ± 0.26	0.03 ± 0.02	0.11 ± 0.06
Unknown 1	1213	0.22 ± 0.19	0.01 ± 0.03	0.06 ± 0.13	0.07 ± 0.1	0.08 ± 0.09	0.04 ± 0.12	0.01	0
*Total percent*		**84.88**	**90.14**	**90.2**	**68.32**	**34.29**	**52.49**	**16.07**	**45.02**
Sesquiterpenes									
* δ*-Elemene^a^	1228	0	0 ± 0.01	0.01 ± 0.01	0.02 ± 0.03	0.07 ± 0.07	0.26 ± 0.3	0.02 ± 0.04	0.09 ± 0.25
Unknown 2	1345	0	0	0.02 ± 0.03	0.02 ± 0.05	0.69 ± 0.47	2.01 ± 2.23	0.01 ± 0.03	0.12 ± 0.36
* α*-Cubebene	1358	0	0	0.01 ± 0.02	0.34 ± 0.31	0.09 ± 0.13	0.14 ± 0.19	0.83 ± 0.15	0.44 ± 0.36
Isoleudene	1381	0.04 ± 0.08	0.01	0.01 ± 0.02	0.07 ± 0.1	0.07 ± 0.08	0	0.11 ± 0.11	0.14 ± 0.37
* α*-Copaene^a^	1386	1.02 ± 0.93	1.02 ± 1.17	0.25 ± 0.25	7.5 ± 6.05	2.6 ± 1.33	5.03 ± 3.2	13.05 ± 2.15	11.67 ± 7.56
Daucene	1389	0	0	0.03 ± 0.05	0.01 ± 0.03	0.04 ± 0.08	0.1 ± 0.2	0	0
* β*-Bourbonene	1395	0.29 ± 0.33	0.17 ± 0.21	0.29 ± 0.34	1 ± 1.06	1.89 ± 1.17	0.67 ± 0.57	2.03 ± 0.68	1.84 ± 1.05
Unknown 3	1398	0.02 ± 0.06	0.02 ± 0.05	0.02 ± 0.03	0.34 ± 0.36	0.48 ± 0.56	0	0.89 ± 0.37	0.41 ± 0.37
Cycloseychellene	1409	0.17 ± 0.32	0.06 ± 0.1	0	0.2 ± 0.39	0.1 ± 0.15	0.56 ± 0.67	0.65 ± 0.38	0.53 ± 0.65
Unknown 4	1420	1.35 ± 2.11	0.53 ± 0.59	0.18 ± 0.19	1.51 ± 1.64	1.17 ± 0.76	4.88 ± 3.81	4.37 ± 0.89	4.27 ± 3.11
* β*-Caryophyllene^a^	1429	0.49 ± 0.68	0.31 ± 0.36	1 ± 1.05	2.41 ± 2.41	11.61 ± 7.41	1.32 ± 1.31	6.77 ± 1.46	2.18 ± 1.42
Unknown 5	1437	0.1 ± 0.16	0.06 ± 0.1	0	0.27 ± 0.4	0.11 ± 0.09	0.74 ± 0.58	1.16 ± 0.66	0.87 ± 0.81
Muurola-3.5-diene	1441	0.51 ± 0.77	0.35 ± 0.42	0.53 ± 0.58	1.92 ± 1.89	6.4 ± 4.37	2.12 ± 1.72	5.73 ± 1.19	2.38 ± 1.36
Himachalene	1444	0.18 ± 0.2	0.14 ± 0.13	0.18 ± 0.23	0.3 ± 0.3	0.77 ± 0.58	0.51 ± 0.61	0.78 ± 0.24	0.42 ± 0.45
Geranyl acetone^a^	1453	0.2 ± 0.31	0.07 ± 0.09	0.08 ± 0.09	0.2 ± 0.17	0.07 ± 0.17	0.69 ± 0.97	0.37 ± 0.39	0.54 ± 0.44
(Z)-Cadina-1(6).4-diene	1456	0.11 ± 0.18	0.09 ± 0.11	0.19 ± 0.23	0.58 ± 0.61	2.42 ± 1.62	0.33 ± 0.39	1.72 ± 0.44	0.55 ± 0.4
Unknown 6	1465	3.72 ± 4.79	2.29 ± 2.72	0.3 ± 0.52	6.39 ± 7.74	9.07 ± 5.02	17.38 ± 13.9	22.93 ± 4.96	13.14 ± 7.86
Unknown 7	1470	0.31 ± 0.4	0.1 ± 0.16	0	0.99 ± 2.16	0.22 ± 0.25	1.09 ± 1.3	1.49 ± 0.73	0.72 ± 0.83
* γ*-Muurolene	1486	0.06 ± 0.16	0.04 ± 0.08	0.03 ± 0.05	0.12 ± 0.19	0.31 ± 0.23	0.21 ± 0.42	0.46 ± 0.4	0.53 ± 0.96
Germacrene D^a^	1492	0.83 ± 0.93	0.67 ± 0.86	1.63 ± 1.84	5.09 ± 4.58	20.03 ± 13.53	2.38 ± 2.35	15.5 ± 6.86	7.42 ± 8.48
Bicyclogermacrene	1508	0.06 ± 0.14	0.07 ± 0.11	0.1 ± 0.17	0.53 ± 0.65	2.48 ± 1.85	2.21 ± 2.21	1.47 ± 0.88	0.47 ± 0.55
Unknown 8	1516	0.14 ± 0.23	0.02 ± 0.05	0.01 ± 0.02	0.1 ± 0.09	0.09 ± 0.08	0.43 ± 0.37	0.43 ± 0.21	0.38 ± 0.31
Unknown 9	1522	0.01 ± 0.04	0.01 ± 0.03	0	0.03 ± 0.05	0	0.15 ± 0.33	0.45 ± 0.93	0.13 ± 0.21
Unknown 10	1524	0.23 ± 0.26	0.15 ± 0.2	0 ± 0.01	0.23 ± 0.35	0.39 ± 0.37	0.5 ± 0.48	0.85 ± 0.65	0.61 ± 0.76
* δ*-Cadinene	1532	0.01 ± 0.02	0.03 ± 0.03	0.02 ± 0.04	0.38 ± 0.35	0.53 ± 0.44	0.92 ± 1.07	1.32 ± 0.81	1.06 ± 1.09
* α*-Calacorene	1553	0.06 ± 0.18	0.02 ± 0.05	0.01 ± 0.02	0.06 ± 0.13	0.05 ± 0.06	0	0.06 ± 0.11	0.32 ± 0.79
* β*-Calacorene	1568	0.02 ± 0.02	0.03 ± 0.06	0.01 ± 0.03	0.06 ± 0.09	0.04 ± 0.08	0.1 ± 0.18	0.02 ± 0.04	0.15 ± 0.18
Total percent		**9.93**	**6.28**	**4.92**	**30.67**	**61.8**	**44.74**	**83.47**	**51.37**

Compounds detected are divided into four groups based on general biosynthetic origin. Within each group, compounds are listed in order of their retention index (RI). For each variety, the mean (± standard errors) of percentages of the VOCs per individual are presented. N, number of individual trees used for scent extraction. *Pollinator-attractive VOCs. Fur. Furanoid, Pyr. Pyranoid. ^a^identified with synthetic reference. The total percentage of each chemical group for each variety is indicated in bold.

### Comparison among varieties of the VOCs emitted by receptive figs

**Figure 2:**
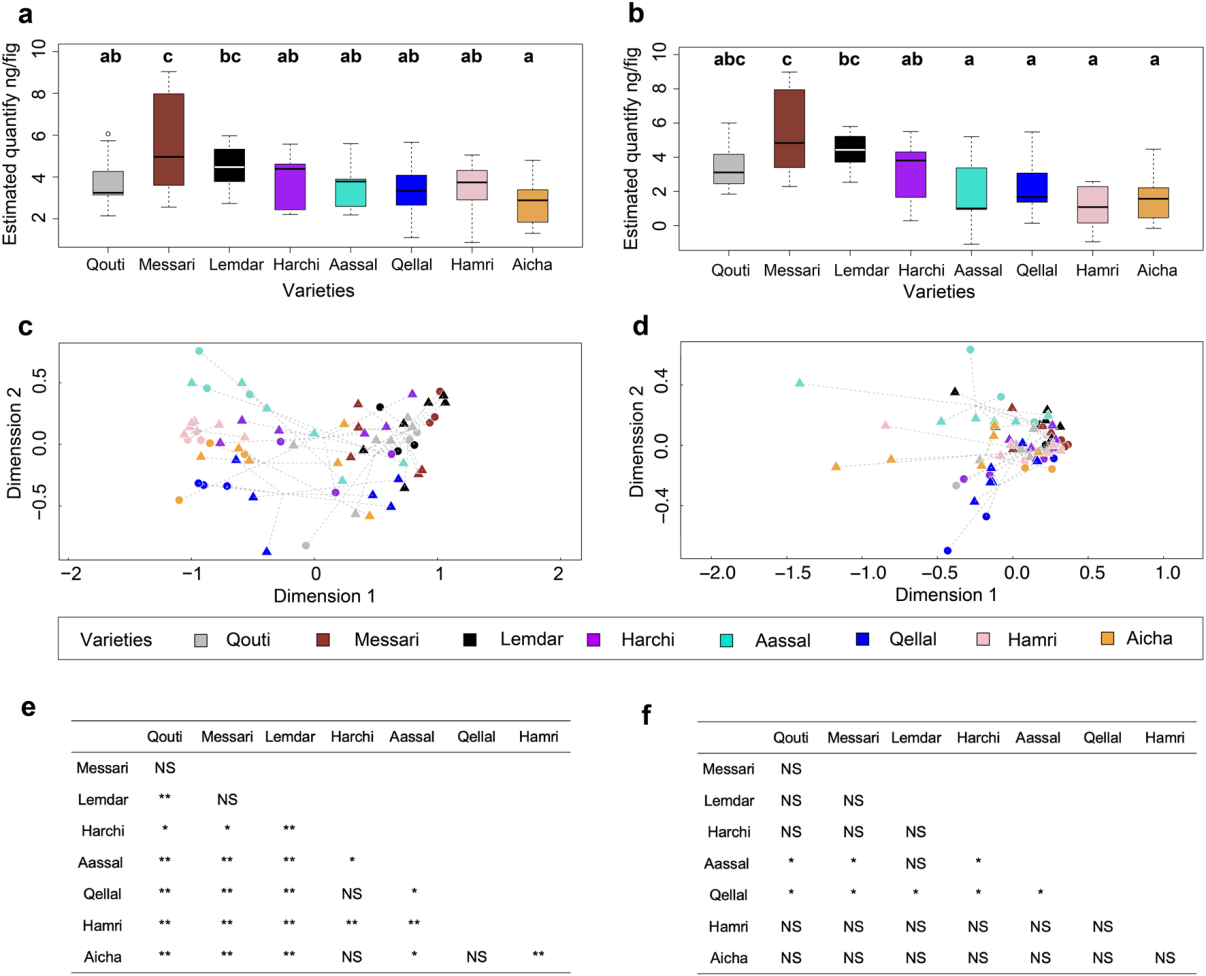
Comparison among the eight varieties of *Ficus carica* of the total estimated quantities and relative proportions of volatile organic compounds (VOCs) emitted by receptive figs. The box plot of estimated total quantifies (log-transformed) of all VOCs (**a**) and of the four pollinator-attractive VOCs (**b**) are presented, together with the results of pairwise comparisons of estimated total quantities within each variety using least-squares means comparisons. Different letters indicate statistically significant differences. Non-metric multidimensional scaling ordination (NMDS) of the relative proportions based on Bray–Curtis distance are presented for all VOCs (**c**, Stress = 0.113) and for the four pollinator-attractive VOCs (**d**, Stress = 0.066) (circle: 2017, triangle: 2018). In addition, results of pairwise comparisons of the relative proportions using PERMANOVA are presented for all VOCs (**e**) and for the four VOCs responsible for pollinator attraction (**f**). *p* < 0.01: _**_p < 0.05: _*_p > 0.05: NS.

Variety, year and their interaction all had significant effects on the total estimated quantity of all VOCs (respectively F_7,70_ = 4.63, *p* < 0.001; F_1,70_ = 11.19, *p* < 0.001; F_7,70_ = 2.25, *p* < 0.05, Fig. 2a). Similarly, variety and its interaction with year had significant effects on the estimated quantity of the four attractive VOCs (F_7,70_ = 8.49, *p* < 0.001; F_7,70_ = 2.73, *p* < 0.05) (Fig. 2b), but year had no significant effect on these quantities (F_1,70_ = 3.62, *p* = 0.06, Fig. 2b). For all VOCs, receptive figs of Messari emitted significantly higher quantities of VOCs than did those of the other varieties (pairwise comparisons *p* < 0.05 for all tests) except Lemdar (*p* = 0.21), and Lemdar emitted significantly higher quantities of VOCs than Aicha (pairwise comparison *p* < 0.05) (Fig. 2a). For the dataset on the four pollinator-attractive VOCs, receptive figs of the varieties Aassal, Hamri, Qellal and Aicha emitted significantly lower quantities than those of Messari and Lemdar (pairwise comparisons *p* < 0.05 for all tests), and Harchi emitted similar quantities as Lemdar and Qouti but lower quantities than Messari (pairwise comparisons *p* < 0.05) (Fig. 2b).

In addition, the relative proportions of VOCs varied significantly among the varieties, between years, and with their interaction for all emitted VOCs (respectively F_7,70_ = 8.87, *p* < 0.001; F_1,70_ = 5.93, *p* < 0.001; F_7,70_ = 2.02, *p* < 0.001, Fig. 2c). For the four attractive VOCs, whereas relative proportions varied significantly among varieties and between years, there was no significant effect of the interaction of these two factors (respectively F_7,70_ = 4.23, *p* < 0.001; F_1,70_ = 4.63, *p* < 0.05; F_7,70_ = 1.21, *p* = 0.28, Fig. 2d). For all VOCs emitted, the volatile profiles of receptive figs were significantly different, except between: Aicha, Harchi and Qellal; Harchi and Qellal; and Messari, Qouti and Lemdar (Fig. 2e). In contrast, for the four attractive VOCs, most varieties had a similar chemical profile, except for Aassal, whose profile was significantly different from those of half of the varieties; and Qellal, whose profile was different from those of all varieties except Hamri and Aicha (Fig. 2f) (Supplementary Fig. S2).

### Comparison among varieties between chemical and genetic distances

Analysis of 80 fig trees (10 per variety) using 15 SSR loci revealed 62 distinct SSR profiles. A total of 53 alleles were detected, with an average of 3.5 alleles per locus (Supplementary Table S1). The highest number of alleles (seven alleles) was detected at the FSYC01 locus, while the lowest number (two alleles) was obtained at five loci (MFC11, MFC8, LMFC26, MFC4 and LMFC19). The size of the alleles varied from 96 bp at the MFC3 locus to 332 at the LMFC19 locus (Supplementary Table S1).

**Figure 3:**
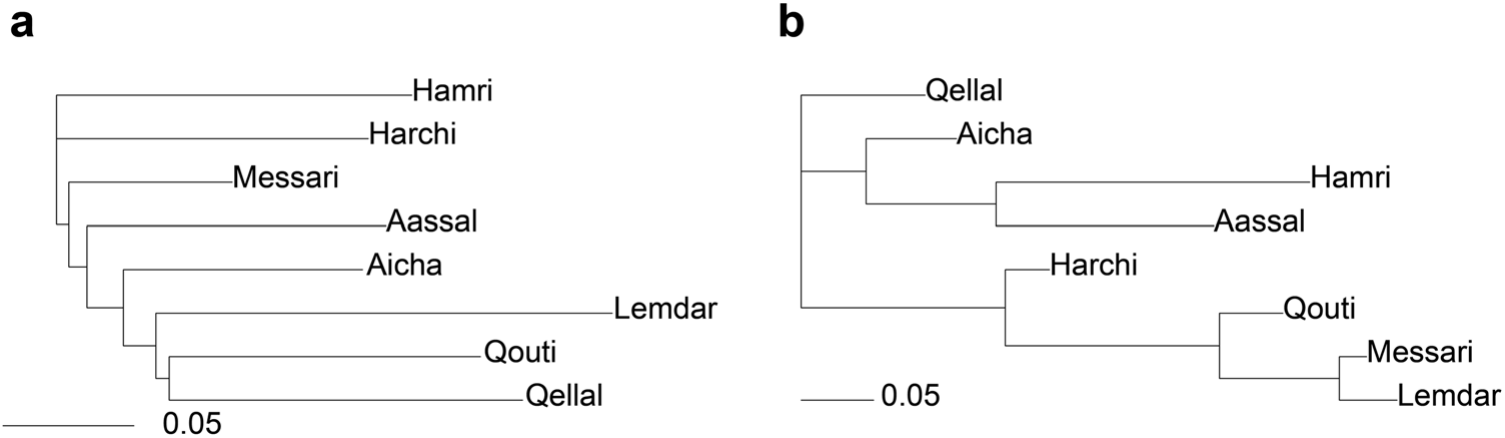
Genetic (**a**) and chemical (**b**) dendrograms. Independent matrices coding genetic distances between varieties were calculated from allele frequency data by neighbor-joining based on Nei distance. Chemical dendrogram was created by using neighbor joining based on the Bray–Curtis distance matrix for the averaged relative proportions of VOCs per variety.

Regarding intra-varietal genetic diversity (Table 2), the level of polymorphism revealed by the 15 SSR markers was relatively high, with an average polymorphism index (*P*) of the order of 0.66 ± 0.07. Thus, the allelic richness per variety (*A*_*P*_) 30 (Lemdar) with an average value of genotypes per variety (*N*_*GP*_) of around 20.25 ± 2.66 and average alleles per locus (*N*_*A*_) of the order of 1.83 ± 0.13. Expected heterozygosity (*H*_*E*_) varied from 0.25 (Hamri) to 0.35 (Qellal and Aassal), with an average value of 0.30 ± 0.04. However, the observed heterozygosity (*Ho*) varied between 0.35 (Hamri) and 0.59 (Aassal), with an average value of 0.49 ± 0.1. For all the varieties, the *Fis* value was negative, indicating an excess of heterozygotes. The genetic distances of Nei calculated from mean values of the allele frequencies were used to produce a hierarchical classification (Fig. 3). The dendrogram groups together six main groups at the threshold of 0.45. These results reflect a significant level of diversity within a relatively restricted geographical area.

**Table 2:** Genetic diversity for the eight varieties of *Ficus carica* based on 15 microsatellite markers.

	P	N_A_	A_P_	N_GP_	H_E_	H_O_	Fis	*p* value
Qouti	0.67	1.87	28.00	18.00	0.32	0.55	− 0.68	** < 0.001**
Messari	0.53	1.60	24.00	18.00	0.26	0.45	− 0.72	** < 0.001**
Lemdar	0.73	2.00	30.00	25.00	0.27	0.36	− 0.30	** < 0.001**
Harchi	0.60	1.87	28.00	21.00	0.30	0.48	− 0.57	** < 0.001**
Aassal	0.67	1.87	28.00	18.00	0.35	0.59	− 0.68	** < 0.001**
Qellal	0.73	1.93	29.00	22.00	0.35	0.56	− 0.61	** < 0.001**
Hamri	0.65	1.74	26.00	22.00	0.25	0.35	− 0.34	** < 0.001**
Aicha	0.67	1.73	26.00	18.00	0.33	0.57	− 0.70	** < 0.001**
Mean	0.66	1.83	27.38	20.25	0.30	0.49	− 0.58	** < 0.001**
Standard deviation	0.07	0.13	1.92	2.66	0.04	0.10	0.17	** < 0.001**

Polymorphism information content (*P*), average number of alleles per locus (*NA*), allelic richness (*A*_*P*_), range size of alleles by variety, number of genotype (*N*_*GP*_), expected heterozygosity (*H*_*E*_), observed heterozygosity (*H*_*0*_) and Wright’s fixation indices (*Fis*) are presented. Significant* p* values (*p* < 0.05) are indicated in bold.

The genetic and chemical dendrograms using respectively the allele frequencies at 15 microsatellite loci and the mean relative proportions of all VOCs (from 2017 and the middle of the manual pollination period of 2018) are presented in Fig. 3. We found no significant correlation (Mantel statistic r = − 0.0731, *p* = 0.609) between genetic and chemical distances in the eight varieties in *F. carica*.

### Variation of VOC emission along the manual pollination period in the different varieties

Despite the fact that an individual tree of *F. carica* bears figs that are on the whole at the same stage of development, there are small differences among figs on the same tree. This is why in the present study we managed to collect receptive figs for all varieties at three different times during the whole period of manual pollination (Fig. 1).

**Figure 4:**
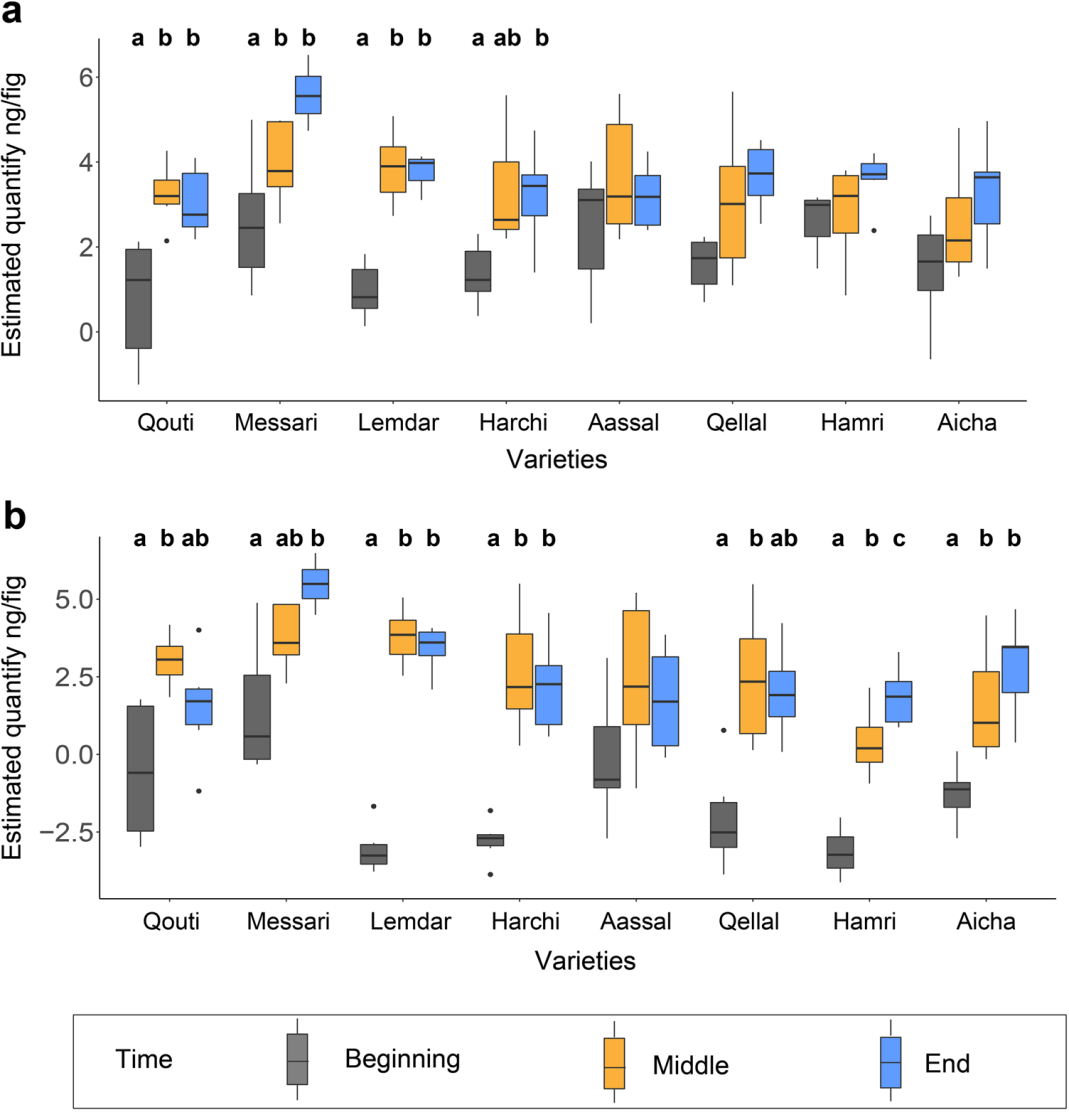
Comparison of the estimated quantities of all volatile organic compounds (VOCs) (**a**) and of the four VOCs responsible for pollinator attraction (**b**) at the three different times within the manual pollination period for each of the eight varieties. Data were log-transformed. Results of pairwise comparisons of estimated total quantifies within each variety using least-squares means comparisons are presented, with different letters indicating statistically significant differences.

The estimated quantity of all VOCs varied significantly among the different times of manual pollination (Fig. 1) for the early and middle-pollinated varieties except Aassal (*p* < 0.05), but not for the three late-pollinated varieties (Table 3). For the four varieties with fluctuating quantities, in pairwise comparisons, the estimated quantity was smaller at the beginning compared to the middle and the end of the manual pollination (Fig. 4a). In addition, figs of the variety Messari released higher quantities of all VOCs at the end of the manual pollination period than at the beginning and the middle of the manual pollination period (Fig. 4a).

**Table 3:** Global results of the comparison among pollination periods of the total estimated quantity and on the relative proportions, of all volatile organic compounds (VOCs) and of the four pollinator-attractive VOCs emitted by figs of each variety.

Varieties	Total estimated quantity					Relative proportion				
	Df	All VOCs		Four VOCs		Df	All VOCs		Four VOCs	
		F	*p* value	F	*p* value		F	*p* value	F	*p* value
Qouti	2, 14	9.11	** < 0.01**	5.60	**0.02**	2, 17	2.99	**0.01**	4.29	**0.02**
Messari	2, 13	10.28	** < 0.01**	11.35	** < 0.01**	2, 16	3.79	** < 0.001**	6.75	** < 0.001**
Lemdar	2, 14	33.25	** < 0.001**	148.36	** < 0.001**	2, 17	13.58	** < 0.001**	9.84	** < 0.001**
Harchi	2, 14	5.33	**0.02**	17.82	** < 0.01**	2, 17	4.7	** < 0.001**	10.72	** < 0.001**
Aassal	2, 14	1.46	0.27	2.72	0.1	2, 17	2.29	**0.03**	2.26	0.1
Qellal	2, 14	1.76	0.21	9.43	** < 0.01**	2, 17	5.48	** < 0.001**	11.78	** < 0.001**
Hamri	2, 14	1.30	0.3	42.73	** < 0.001**	2, 17	4.17	** < 0.001**	3.06	**0.04**
Aicha	2, 13	2.99	0.09	10.36	** < 0.01**	2, 16	3.52	**0.01**	1.13	0.35

For the estimated quantity, analysis of deviance was performed on a linear regression model of VOCs (log-transformation). For the relative proportions, permutational multivariate analysis of variance (PERMANOVA) performed on proportions of VOCs (standardization prior to the analysis). Degrees of freedom (Df) and results of the test (F) are indicated. Significant *p* values (*p* < 0.05) are indicated in bold.

The total estimated quantity of the four attractive VOCs varied significantly among the different times of the manual pollination period for all varieties except Aassal (Table 3, *p* < 0.05). In pairwise comparisons, in most varieties, figs emitted lower quantities of these four VOCs at the beginning compared to the middle and end of pollination (Fig. 4b). For these two last times, the quantity emitted was similar in all varieties except for Hamri and Messari, for which the quantity at the end was higher than in the middle. For the varieties Qellal and Qouti, the quantity of VOCs emitted at the middle of the pollination period was higher than at the beginning and end.

Regarding the relative proportions of all VOCs, manual pollination time had a significant effect for all varieties (Table 3, PERMANOVA, *p* < 0.05). In pairwise comparisons, different patterns of variation among varieties were found but generally the relative proportions at the beginning of manual pollination were significantly different from those in the other times, except for Aassal, Qellal and Qouti (Supplementary Fig. S4). For Aassal, there was no significant difference among the three times. For Qellal, the middle period was only significantly different from the beginning. For Qouti, the middle period was significantly different from the two other periods.

Manual pollination time had a significant effect on the relative proportions of the four chemical classes (benzenoid, fatty acid, monoterpene and sesquiterpene biosynthetic pathways) for all varieties (Supplementary Table S2, *p* < 0.05), with pairwise comparisons showing different patterns of variation among varieties (Supplementary Fig. S5).

**Figure 5:**
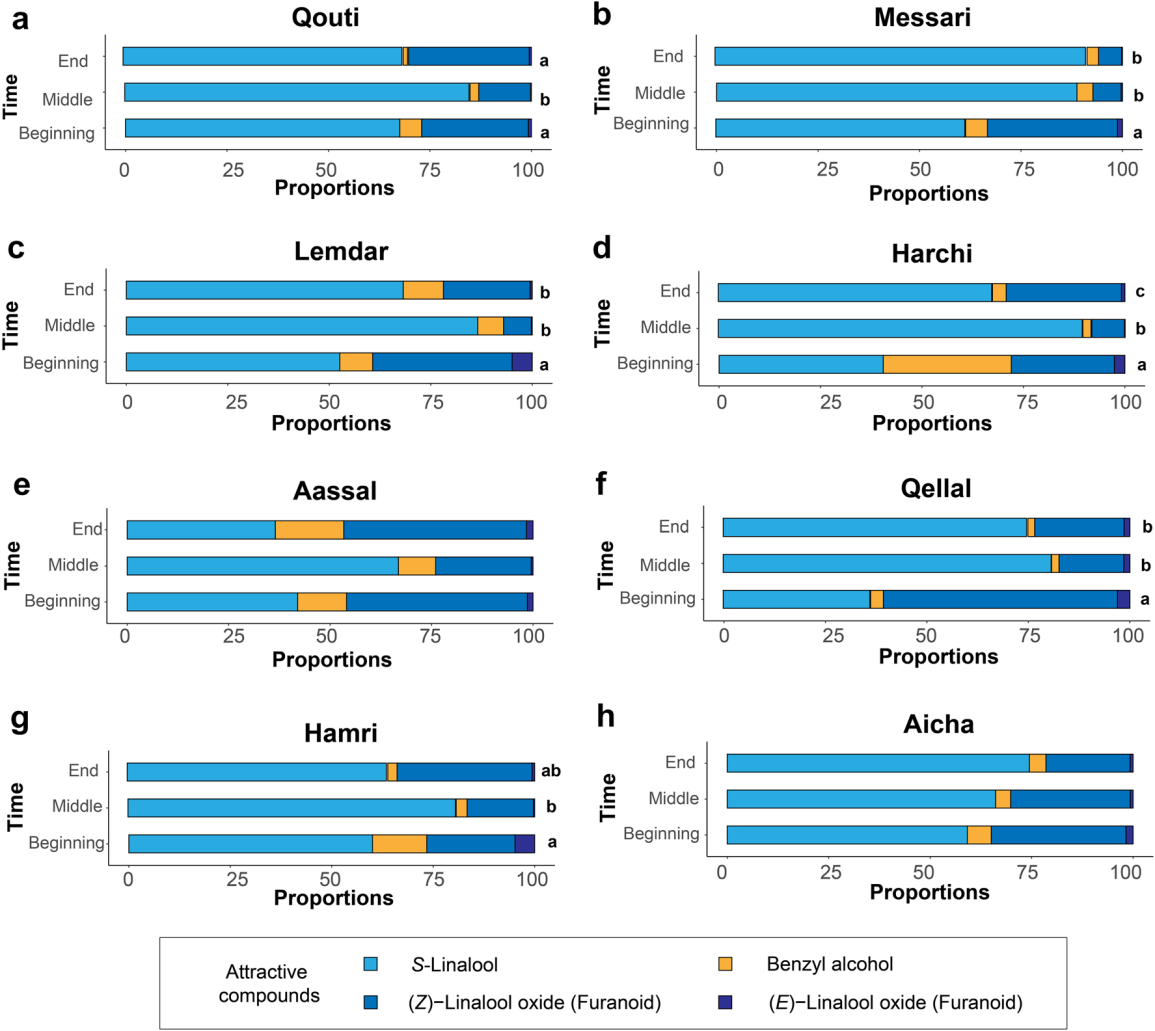
Relative proportions of the four pollinator-attractive volatile organic compounds (VOCs) at the three different time within the manual pollination period of the eight varieties (**a**, **b**, **c**, **d**, **e**, **f**, **g** and **h**). Results of pairwise comparisons between times were performed with correction of *p*-values using the “fdr” procedure. Different letters indicate statistically significant differences. When no letter is indicated, the global test was not statistically significantly different.

Relative proportions of the four attractive VOCs collected at different time during the manual pollination season were significantly different for all varieties except Aassal and Aicha (Table 3, *p* < 0.05, Fig. 5). For the six other varieties, pairwise comparisons showed that proportions of these four VOCs at the beginning of the manual pollination period were significantly different from those in the other times, except for Hamri, where proportions at the beginning were significantly different only from those at the end, and for Qouti, where proportions at the middle were significantly different from those in the other times (Fig. 5). For Harchi, differences in the proportions of these VOCs were different among all three times (Fig. 5d).

## Discussion

In the present study, through the characterization of the volatile profiles of the receptive figs of eight *F. carica* varieties from northern Morocco, we showed substantial inter-varietal differences. Some varieties (Qouti, Lemdar and Messari) release high amounts of monoterpenes, while others (Aassal, Aicha, Hamri and Qellal) emit high amounts of sesquiterpenes, and one variety is intermediate (Harchi). In addition, we found inter-varietal variations in total amounts and relative proportions of VOCs, as well as in temporal patterns of VOC emission. However, despite these inter-varietal differences, the chemical signal responsible for pollinator attraction is highly conserved between varieties.

The dioecious Mediterranean fig tree, *F. carica*, has a particular phenology with generally male trees flowering twice a year, in spring and summer, and female trees flowering only once a year, in summer, and therefore partially synchronously with males in summer. Previous studies have described the VOCs emitted by receptive figs of wild female and male trees of *F. carica*^23,29,30^, but not those emitted by cultivated varieties. These studies reported that the relative proportions of all VOCs emitted by receptive male figs in spring differed significantly from those of receptive male and female figs produced in summer, with higher relative proportions of several sesquiterpenes in summer in both male and female figs compared to spring males^30^. The authors of these studies hypothesized that this seasonal difference in VOC emission could be related to abiotic factors such as hydric stress and temperature. Indeed, it is known that while emission by plants of monoterpenes, such as linalool, can decrease under drought conditions, emission of sesquiterpenes, such as *β*-caryophyllene, can increase under high temperature^31^. Also, high temperature has generally been found to increase the rates of total emissions of VOCs in plants^32,33^. Interestingly, in *F. carica* studied in southern France, the relative proportions of the four VOCs emitted at receptivity to attract the specific pollinator were not significantly different between spring male figs and summer male and female Figs^23^. However, the absolute quantity of these four VOCs was significantly higher for male figs in spring than for male and female figs in summer^23^. It has been suggested that this quantitative inter-seasonal difference in VOC emission by receptive figs -which is in the opposite direction to the difference generally driven by temperature- could be the result of pollinator-mediated selection: because the density of pollinators is much lower in spring than in summer^22,29^, spring male figs should be selected to emit a greater quantity of VOCs to attract pollinators^23^.

In the present study, the chemical profiles of the receptive figs of the early (Qouti) and three of the middle-pollinated varieties (Messari, Lemdar and Harchi) (Fig. 1) were closer to that already described^23^ for wild spring male figs than to that of wild females (greater relative proportions of monoterpenes than sesquiterpenes, Table 1 and Supplementary Fig. S1). On the contrary, Aassal and the three late-pollinated varieties (Fig. 1) presented a “classical” volatile profile of wild female figs (greater relative proportions of sesquiterpenes compared to monoterpenes, Table 1 and Supplementary Fig. S1). These inter-varietal differences in the emission of all VOCs, for both total estimated quantity and relative proportions, could be driven by abiotic factors, human driven-selection, or both combined. However, in this study, we can reject the hypothesis that abiotic factors affected emission of VOCs, as all individuals studied were cultivated on the same site, thus exposed to similar environmental conditions. In addition, although slight variations in micro-climatic conditions may have occurred, we found that (1) ambient temperature during the collection phases was very homogeneous (Fig. 1) and that the temperature registered inside the sample bag had no significant effect on the total quantity emitted on the relative proportions of all VOCs, or on the relative proportions of the four attractive VOCs, for all varieties. Therefore, the inter-varietal differences found in this study were most likely related to whether they were pollinated early or late in the season, and therefore variation results mainly from human-driven diversification. We hypothesize that the varieties emitting the highest quantities of the VOCs responsible for pollinator attraction (Messari, Qouti and Lemdar) are more effective at attracting pollinators early in the flowering season when pollinator density is low. These female varieties with very different chemical profiles from wild females were probably selected during the domestication process because their pollination early in the season was facilitated by their high VOC emissions. Indeed, efficient pollination of figs increases the yield and quality of the fruits^34^.

Compared to the other VOCs emitted by receptive figs, in these eight varieties, the relative proportions of the four VOCs that attract the pollinator are relatively homogeneous, except for Aassal and Qellal (Table 1, Figs. 2, S2). Despite this small variation, the proportions of the four VOCs are still within the range that ensures pollinator attraction^23^. Indeed, for all the varieties studied here, fruit production requires attracting female fig wasps. Thus, our results seem to indicate that in *F. carica*, stabilizing selection during human-driven varietal diversification has maintained the chemical signal responsible for pollinator attraction. This hypothesis could be tested by comparing the chemical profiles of varieties that do not require the presence of pollinators (*i.e.* parthenocarpic varieties) and those that depend exclusively on pollinators for fruit production.

In the genetic analyses, the difference we found between expected and observed heterozygosity is typical for clonally propagated entities. Indeed, clonal propagation in domesticated plants can result in an excess of heterozygosity for several reasons^35^. First, highly heterozygous genotypes may have been selected by humans for clonal propagation. Also, clonal lineages accumulate mutations, and since it is unlikely that the same mutation occurs at the same locus on both chromosomes, the clonal lineage becomes more heterozygous over time. For instance, it has been shown in manioc that domestication by clonal propagation has resulted in the fixation of deleterious mutation^36^. As *F. carica* is an emblematic plant of the Mediterranean region with a large number of varieties and a long history of domestication^21^, it would be interesting to study the consequences of human driven-selection on the genetics of these different varieties in order to shed light on the evolutionary trajectories of this species under domestication.

The lack of correlation between chemical and genetic distances is probably not surprising, as neutral markers were used in the present study, but correlation could occur at the level of biosynthetic pathways^37^, or when specific loci are selected. For instance, in *Cannabis sativa* and *C. indica*, differences between cultivars are driven by terpene synthase genes on different chromosomes, which control the concentrations of terpenes in this species^38^. Terpene synthase genes involved in the synthesis of some monoterpenes (including linalool) and sesquiterpenes in *F. carica* figs have been identified^39^. In the present study, since significant differences in the emission of mono- and sesquiterpenes have been shown between some varieties (Table 1, Supplementary Fig. S1), comparison of the expression of these genes in receptive figs of these varieties would be interesting in exploring the links between chemical and genetic differences.

Our results revealed that in the eight varieties studied, the total estimated amount of all VOCs and the four attractive VOCs changed at different times during the manual pollination period. As different figs were collected for each sampling time, we cannot know whether there are quantitative or qualitative variations in VOC emission (1) for a single fig along the whole receptive stage or (2) among different figs of the same individual tree. Nevertheless, in wild male and female fig trees, receptivity lasts 2–3 weeks if the figs are not pollinated^40^ and variation in the VOC emission along the receptive period has been described^29^. In other plants, floral VOC emission is known to be developmentally regulated and displays variable patterns: emission increasing before the flowers are ready for pollination and either decreasing or remaining relatively constant during flower receptivity^41,42^. In the future, it would be interesting to compare the VOC emission patterns of individual figs along the receptive stage between varieties and wild individuals of *F. carica* to examine whether human-driven selection may have affected these patterns.

For most of the varieties studied here, during the manual pollination times, the total estimated quantity of the four attractive VOCs was generally higher at the middle and end compared to the beginning. Therefore, for most varieties, the best time for pollination would be the middle of the manual pollination period and this is when most manual pollination takes place (Fig. 1). Nevertheless, farmers start manual pollination very early for some varieties, possibly because all varieties need to be pollinated during a short period. For practical reasons of availability of male figs, this pollination must be spread out over time during this period. Thus, manual pollination is controlled^28^ and repeated two to three times for each tree during this period to enhance the pollination of figs until these are considered fully pollinated (Fig. 1).

Three varieties (Aassal, Qouti and Messari) show a pattern of temporal variation different from the others. For these three varieties, the quantity of VOCs emitted at the beginning of the manual pollination period is already high, which can explain the fact that farmers start pollination with Qouti. Usually, Messari is manually pollinated at the beginning of the season too, but the year we conducted our sampling, farmers decided to start pollinating this variety in the middle of the season (Fig. 1). For these three varieties, as the period of manual pollination does not correspond to the peak of VOC emission, reasons other than the intensity of the chemical signal must also contribute to explaining the farmers’ pollination strategy.

## Materials and methods

### Study site and plant materials

The sample collection took place in the village of Talandaoued (34.83124 North, 5.0757267 West), part of the rural commune of Bni Ahmed Charqia (Chefchaouen, Morocco), a mountainous region of the western Rif belonging to the Jbala country (Mountain Arabic-speaking socio-cultural group). The region is characterized by hills, mountains and plains. The climate is of the Mediterranean type, with a wet winter and a dry summer. The average annual temperature is 18.2 °C and the average annual precipitation is 512 mm. Choice of the study site is justified by the significant agro-diversity of fig trees and the persistence of the practice of manual pollination^27,28^. Thus, it is characterized, within an area of eight hectares, by the presence of 15 locally-identified varieties of fig tree (*Ficus carica* L.) of which only eight are non-parthenocarpic and require manual pollination (caprification) at the receptive stage to ensure fruit production. In this study, we only considered these eight varieties, no wild plants, and relied on farmers’ knowledge for receptive periods and timing of the manual pollination, as this may vary from year to year according to climate. The manual pollination lasted around five weeks in total and was spread out during this period between the varieties that bear most of their receptive figs early (Qouti, Messari), at the middle (Harchi, Aassal and Lemdar) or late (Aicha, Hamri and Qellal) in the season (Fig. 1). During this season, after the first manual pollination, the trees of each variety were pollinated two to three times at an interval of about five days. In 2018, manual pollination of Messari was only conducted at the middle of the manual pollination period. It should be noted that this study complies with the relevant institutional, national and international guidelines and legislation. Thus, the farmers contacted for access to the cultivated plant material gave their agreement to obtain the plant samples as well as the data on manual pollination periods. The Moroccan team involved in this project also obtained the required authorizations from the local village administration.

### Genetic analysis

DNA was extracted from 70 mg of young leaves of the eight varieties (ten trees of each variety, including the trees that were used for odor collection in 2018) using the Dneasy Plant Mini Kit (Qiagen). Among the SSR markers developed^43–45^, we selected 15 loci^26^ (Supplementary Table S1). The microsatellite amplifications were carried out according to the standard Multiplex PCR protocol and using five multiplexes [(LMFC30, LMFC32 and FSYCO4), (MFC2, FSYCO1 and MFC11), (MFC3, MFC1 and LMFC28), (LMFC24, MFC8 and LMFC26) and (MFC9, MFC4 and LMFC19). SSR genotyping was performed in an Abi 3500XL automated capillary sequencer and analyses were performed using Genemapper software (v. 5.0).

### Collection of VOCs

Collection of volatile organic compounds (VOCs) of receptive figs (*i.e.,* ready to be pollinated) was conducted during two successive years on different trees between the two years, in situ using dynamic headspace^46,47^. In July 2017, VOCs were collected from three individuals per variety from eight different fig varieties. In 2018, samples from six individuals per variety were collected to analyze the VOCs of the varieties at three different time during the manual pollination period; (i) at the beginning (when the first manual pollination of the early variety was conducted), (ii) at the middle (when the first manual pollination of the middle varieties was conducted for the first time, corresponding to the same development stage as in the previous year), and (iii) at the end (when the last manual pollination of the late varieties was conducted) (Fig. 1). For each collection, 20 receptive figs per individual were enclosed in a polyethylene terephtalate (Nalophane^®^, Kalle Nalo GmbH, Wursthüllen, Germany) bag of volume around 8 L. Microtraps, ChromatoProbe^®^ quartz microvials (Varian Inc., length: 15 mm; inner diameter: 2 mm), containing 3 mg of a 1:1 mixture of Tenax-TA and Carbotrap (60–80 and 20–40 mesh respectively, Sigma-Aldrich, Munich, Germany) were used as adsorbent traps. One microlitre of a solution of internal standards (*n*-nonane and *n*-dodecane, 110 ng μl^−1^ of each) was added to each trap before scent collection. Bags containing figs were left in the shade for 30 min and during 10 min, air (flow rate 200 ml min^−1^) was drawn out of the bag and through the adsorbent trap for 5 min. In parallel, for each control collection, extractions were performed using empty bags, to control for ambient contaminant compounds. we collected one blank sample per site per collection day. Collections were done under natural light and ambient temperature, between 10:30 and 17:00 h. Data on additional parameters were collected during harvesting, including the temperature at the time of harvesting, inside and outside the collection bags.

### Chemical analysis of VOCs

Traps were stored in the dark in a freezer (− 20 °C) until analysis. Samples were analyzed by gas chromatography-mass spectrometry (GC–MS) using a similar method as Souto-Vilarós et al.^47^. These samples were analyzed by a gas chromatograph (GC, Trace 1310, Thermo Scientific, Milan, Italy) coupled with a mass spectrometer (ISQ QD Single Quadrupole, Thermo Scientific, Milan, Italy) with an Optima 5-MS capillary column (30 m, 0.25 mm ID, 0.25 μm film thickness, Machery-Nagel, Düren, Germany). The injection system composed of a Thermal Desorption Unit (TDU) and a Cold Injection System (CIS) (Gerstell, Mülheim, Germany), absorbent traps were handled with a Multi-Purpose Sampler (Gerstell, Mülheim, Germany). The program for the analysis was as follows: oven held at 40 °C for 3 min, then increased from 40 °C to 220 °C at 5 °C min^−1^, and finally from 220 to 250 °C at 10 °C min^−1^. Temperature was then maintained at 250 °C for 2 min. The transfer line was 250 °C. The ion source of the mass spectrometer was 200 °C. The acquisition was from 38 m*/z* to 350 m/z at 70 eV ionisation energy. Xcalibur TM 266 software (Thermo Scientific TM, Milan, Italy) was used for data processing. Retention index (RI) was calculated by a series of *n*-alkanes (alkanes standard solution, 04070, Sigma Aldrich, Munich, Germany). Compounds were identified based on computer matching of the mass spectra with a library (NIST 2017 MS), and confirmation by comparison of retention index reported in literature^48^. When possible, mass spectra and retention index were compared to those of reference compounds run in the same GC–MS with the same method. Putative contaminant compounds were subtracted from our chromatograms by comparing spectra of each sample with the respective control sample. Trace compounds, which always occurred in mean proportions per variety of less than 0.1%, were excluded from the analyses. In addition, the stereochemistry of linalool of one individual per variety was determined using the same analytical method as indicated above in the same GC–MS equipped with a *β*-cyclodextrin chiral capillary column (Cyclosil-B, 30 m–0.25 mm i.d., 0.25 μm film thickness, Agilent J&W columns, USA).

### Chemical dataset

In order to estimate the quantity of VOCs emitted by a receptive fig of each sample, the calculation was performed using the two internal standards as reference and the following Eq. (1):

1 $$Q_{x} = {{\left( {\left( { \frac{{A_{x} { }}}{{A_{C9} }}} \right) + \left( { \frac{{A_{x} { }}}{{A_{C12} }}} \right)*110} \right)} \mathord{\left/ {\vphantom {{\left( {\left( { \frac{{A_{x} { }}}{{A_{C9} }}} \right) + \left( { \frac{{A_{x} { }}}{{A_{C12} }}} \right)*110} \right)} {2*20}}} \right. \kern-0pt} {2*20}}$$

where Q_x_: estimated quantity of the VOCx (ng fig^−1^); A_x_: peak area of the VOCx, A_C9_: peak area of the *n*-nonane, A_C12_: peak area of the *n*-dodecane. In each sample 110 ng of each internal standard were injected. Twenty figs were used during odor collection.

For the comparison of volatile profiles among different varieties and between years, two estimated-quantity datasets (total of all VOCs and total of the four pollinator-attractive VOCs) were created combining data from 2017 and the middle of manual pollination of 2018. To test the effect of the time within the manual pollination period for each variety separately, two estimated-quantity datasets per variety (all VOCs and four attractive VOCs) were built using all data from 2018.

In addition, for each sample, the percentage relative to the total peak area (i.e. relative proportion), for all VOCs on the one hand and only for the four pollinator-attractive VOCs on the other, of each VOC was calculated. As with the estimated quantity, we generated four datasets for the relative proportions. For the effect of varieties and year, we combined data from 2017 and the middle of the 2018 manual pollination for all VOCs and the four attractive VOCs. To test the effect of time within the manual pollination period on each variety separately, we used all 2018 data. Moreover, we generated a dataset of relative proportions of the VOCs belonging to the different chemical classes (monoterpenes, sesquiterpenes, fatty acid derivatives, and benzenoids) from the eight varieties using the samples from 2018 for all VOCs.

In order to compare chemical and genetic distance, a dataset using allele frequencies among all replicates of each variety was calculated, thus with only one point per variety. Mirroring this, for each variety, the available replicates on the relative proportions of all VOCs (2017 data and the middle of the 2018 manual pollination) were averaged by variety.

### Statistical analyses

All the data on chemical analyses were performed in R (v. 4.2.1;^49^) with the packages emmeans^50^, vegan^51^, RVAideMemorie^52^, ade4^53^ and hierfstat^54^. Estimation of the various parameters of genetic diversity was performed using GENETIX software (v. 4.03) and NTSYSpc software (v. 1.05).

The significant effect of varieties and time of the manual pollination period on the total estimated quantity of VOCs emitted by one fig (for all VOCs and only the four attractive VOCs), was tested using linear regression models including temperature in the bag and year as co-factors, and followed by a deviance analysis. The data were log-transformed prior to the analyses to obtain normality of the residuals. Then pairwise comparisons between varieties were performed computing estimated marginal means for the factor varieties (least-squares means).

For the comparison of the relative proportions of VOCs between varieties, a distance matrix comparing the dissimilarity between all samples in pairs using a Bray–Curtis index was built, on the dataset including all VOCs on the one hand, and on the dataset including only the four attractive VOCs on the other. Data were standardized prior to the analysis. Then, for each dataset the distances between all pairs of samples in the distance matrix were plotted using non-metric multidimensional scaling analysis (NMDS). Effects of varieties were then tested on the distance matrix, including temperature and year in the bag as co-factors, using permutational multivariate analysis of variance (PERMANOVA) with 9999 permutations. If significant effects were found, pairwise comparisons between varieties were performed with correction of *p*-values using the false discovery rate (fdr) method^55^. Similar analysis was used on the datasets of relative proportions of all samples from 2018, for all VOCs, for only the four attractive VOCs, and for the relative proportions of four different chemical classes, to test the effect of time within the manual pollination period with temperature as co-factor.

For genetic data, polymorphism was estimated by the average number of alleles per locus (*NA*), allelic richness (*AP*), number of genotypes (*NGP*), polymorphism information content (*P*)^56^, observed heterozygosity (*H0*), expected heterozygosity (*HE*), and Wright's fixation index (*Fis*)^57^. Hierarchical clustering using Nei’s distance^58^ and the UPGMA clustering method was performed. In addition, to compare genetic and chemical distances, we created independent matrices coding the standard Nei genetic distance between varieties, based on allele frequency data, and the Bray–Curtis distance for the average chemical distance of all VOCs. Then, for these two matrices we created dendrograms using the neighbor-joining method^58^. Finally, we tested the correlation between chemical and genetic distances using the Mantel test.

## Supplementary Information

Supplementary Information.

## Data Availability

All data generated or analyzed during this study are included in this published article. The raw data supporting these analyzes will be made available by the authors, without undue reservation.

## References

[1] Purugganan M, Fuller D (2009). The nature of selection during plant domestication. Nature.

[2] Whitehead SR, Turcotte MM, Poveda K (2017). Domestication impacts on plant–herbivore interactions: A meta-analysis. Phil. Trans. R. Soc. B.

[3] Gaillard MDP, Glauser G, Robert CAM, Turlings TCJ (2018). Fine-tuning the ‘plant domestication-reduced defense’ hypothesis: Specialist vs generalist herbivores. New Phytol..

[4] McKey D, Cavagnaro TR, Cliff J, Gleadow R (2010). Chemical ecology in coupled human and natural systems: People, manioc, multitrophic interactions and global change. Chemoecology.

[5] Wu Y (2019). Allelochemicals targeted to balance competing selections in African agroecosystems. Nat. Plants.

[6] Bradbury EJ, Emshwiller E (2011). The role of organic acids in the domestication of *Oxalis tuberosa*: A new model for studying domestication resulting in apposing crop phenotypes. Econ. Bot..

[7] Fernandes NS, Silva FAN, Aragão FAS, Zocolo GJ, Freitas BM (2019). Volatile organic compounds role in selective pollinator visits to commercial melon types. J. Agric. Sci..

[8] Saunier A (2022). Lavender sensitivity to water stress: Comparison between eleven varieties across two phenological stages. Ind. Crop. Prod..

[9] Rodriguez-Saona C, Parra L, Quiroz A, Isaacs R (2011). Variation in highbush blueberry floral volatile profiles as a function of pollination status, cultivar, time of day and flower part: Implications for flower visitation by bees. Ann. Bot..

[10] Klatt BK, Burmeister C, Westphal C, Tscharntke T, Fragstein MV (2013). Correction: Flower volatiles, crop varieties and bee responses. PLoS ONE.

[11] Klein AM (2007). Importance of pollinators in changing landscapes for world crops. Proc. R. Soc. B..

[12] Guitton Y (2018). A comparative study of terpene composition in different clades of the genus *Lavandula*. Bot. Lett..

[13] Pokajewicz K (2022). Comparative evaluation of the essential oil of the new Ukrainian *Lavandula angustifolia* and *Lavandula x intermedia* cultivars grown on the same plots. Molecules.

[14] Vainstein A, Lewinsohn E, Pichersky E, Weiss D (2001). Floral fragrance. New inroads into an old commodity. Plant Physiol..

[15] Fattorini R, Beverley J, Glover BJ (2020). Molecular mechanisms of pollination biology. Annu. Rev. Plant Biol..

[16] Dormont L, Joffard N, Schatz B (2019). Intraspecific variation in floral color and odor in Orchids. Int. J. Plant Sci..

[17] Dar MS (2021). Influence of domestication on specialized metabolic pathways in fruit crops. Planta.

[18] Mostafa S, Wang Y, Zeng W, Jin B (2022). Floral scents and fruit aromas: Functions, compositions, biosynthesis, and regulation. Front. Plant Sci..

[19] Du F (2019). Volatile composition and classification of *Lilium* flower aroma types and identification, polymorphisms, and alternative splicing of their monoterpene synthase genes. Hortic. Res..

[20] Shi T (2019). Exploration of floral volatile organic compounds in six typical *Lycoris* taxa by GC-MS. Plants.

[21] Zohary D, Spiegel-Roy P (1975). Beginnings of fruit growing in the old world: Olive, grape, date, and fig emerge as important Bronze Age additions to grain agriculture in the Near East. Science.

[22] Kjellberg F, Gouyon P-H, Ibrahim M, Raymond M, Valdeyron G (1987). The stability of the symbiosis between dioecious figs and their pollinators: a study of *Ficus carica* L. and *Blastophaga psenes* L.. Evolution.

[23] Proffit M (2020). Chemical signal is in the blend: bases of plant-pollinator encounter in a highly specialized interaction. Sci. Rep..

[24] Condit IJ (1947). The fig.

[25] Tribolet I (1912). Caprification of smyrna figs. Agricult. J. Union S. Afr..

[26] Achtak H, Ater M, Oukabli A, Santoni S, Kjellberg F, Khadari B (2010). Traditional agroecosystems as conservatories and incubators of cultivated plant varietal diversity: The case of fig (*Ficus carica* L.) in Morocco. BMC Plant Biol..

[27] Hmimsa Y, Aumeeruddy-Thomas Y, Ater M (2012). Vernacular taxonomy, classification and varietal diversity of fig (*Ficus carica* L.) among Jbala cultivators in Northern Morocco. Hum. Ecol..

[28] Aumeeruddy-Thomas Y, Hmimsa Y, Stepanoff C, Vigne J-D (2018). Fig and olive domestication in the Rif, Northern Morocco. Entangled human and tree lives and history. Hybrid Communities Biosocial Approaches to Domestication and Other Transspecies Relationships.

[29] Grison-Pigé L (2001). Limited intersex mimicry of floral odour in *Ficus carica*. Funct. Ecol..

[30] Soler C, Proffit M, Bessière J-M, Hossaert-McKey M, Schatz B (2012). Evidence for intersexual chemical mimicry in a dioecious plant. Ecol. Lett..

[31] Holopainen JK, Gershenzon J (2010). Multiple stress factors and the emission of plant VOCs. Trends Plant Sci..

[32] Farré-Armengol G, Filella I, Llusia J, Peñuelas J (2013). Floral volatile organic compounds: Between attraction and deterrence of visitors under global change. J. Perspect. Plant Ecol. Evol. Syst..

[33] Peñuelas J, Staudt M (2010). BVOCs and global change. Trends Plant Sci..

[34] Trad M, Ginies C, Gaaliche B, Renard CMGC, Mars M (2012). Does pollination affect aroma development in ripened fig *Ficus carica* L. fruit?. Sci. Hortic..

[35] McKey D, Elias M, Pujol B, Duputié A (2010). The evolutionary ecology of clonally propagated domesticated plants. New Phytol..

[36] Ramu P (2017). Cassava haplotype map highlights fixation of deleterious mutations during clonal propagation. Nat. Genet..

[37] Levin R, McDade L, Raguso R (2003). The systematic utility of floral and vegetative fragrance in two genera of *Nyctaginaceae*. Syst. Biol..

[38] Watts S (2021). *Cannabis* labelling is associated with genetic variation in terpene synthase genes. Nat. Plants.

[39] Nawade B (2020). Characterization of terpene synthase genes potentially involved in black fig fly (*Silba adipata*) interactions with *Ficus carica*. Plant Sci..

[40] Khadari B, Hochu I, Santoni S, Kjellberg F (2001). Identification and characterization of microsatellite loci in the common fig (*Ficus carica* L.) and representative species of the genus *Ficus*. Mol. Ecol. Notes.

[41] Dudareva N (2000). Developmental regulation of methyl benzoate biosynthesis and emission in snapdragon flowers. Plant Cell.

[42] Vassao DG (2006). Chavicol formation in sweet basil (*Ocimum basilicum*): Cleavage of an esterified C9 hydroxyl group with NAD(P)H-dependent reduction. Org. Biomol. Chem..

[43] Ahmed S, Dawson DA, Compton SG, Gilmartin PM (2007). Characterization of microsatellite loci in the African fig, *Ficus sycomorus* L. (Moraceae). Mol. Ecol. Notes.

[44] Giraldo E, Viruel MA, López-Corrales M, Hormaza JI (2005). Characterisation and cross-species transferability of microsatellites in the common fig (*Ficus carica* L.). J. Horticult. Sci. Biotechnol..

[45] Khadari B, Gibernau M, Anstett MC, Kjellberg F, Hossaert-McKey M (1995). When figs wait for pollinators: The length of fig receptivity. Am. J. Bot..

[46] Soler C (2011). Geographic variation of floral scent in a highly specialized pollination mutualism. Phytochemistry.

[47] Souto-Vilarós D (2018). Pollination along an elevational gradient mediated both by floral scent and pollinator compatibility in the fig and fig-wasp mutualism. J. Ecol..

[48] Adams RP (2007). Identification of Essential Oil Components by Gas Chromatography/Mass Spectrometry.

[49] R Core Team, n.d. R: A language and environment for statistical computing, in *R Foundation for Statistical Computing, Vienna, Austria*, http://www.R-project.org/ (2013).

[50] Lenth R. Emmeans: Estimated marginal means, aka least-squares means. R package version 1.8.2. https://CRAN.R-project.org/package=emmeans (2022).

[51] Oksanen J. *et al.* Vegan: Community ecology package. R package version 2.6-4. https://CRAN.R-project.org/package=vegan (2022).

[52] Hervé M. RVAideMemoire: Testing and plotting procedures for biostatistics. R package version 0.9-81-2. https://CRAN.R-project.org/package=RVAideMemoire (2022).

[53] Dray S, Dufour A (2007). The ade4 package: Implementing the duality diagram for ecologists. J. Stat. Softw..

[54] Goudet J., Jombart T. Hierfstat: estimation and tests of hierarchical F-statistics. R package version 0.5-11. https://CRAN.R-project.org/package=hierfstat (2022).

[55] Benjamini Y, Hochberg Y (1995). Controlling the false discovery rate: A practical and powerful approach to multiple testing. J. R. Stat. Soc. B.

[56] Anderson JA, Churchill GA, Autrique JE, Tanksley SD, Sorrells ME (1993). Optimizing parental selection for genetic linkage maps. Genome.

[57] Weir BS, Cockerham CC (1984). Estimating F-statistics for the analysis of population structure. Evolution.

[58] Nei M (1987). Molecular Evolutionary Genetics.

