# Supplementary material for: Floral scent of the Mediterranean fig tree: significant inter-varietal difference but strong conservation of the signal responsible for pollinator attraction
Source: Sci Rep. 2023 Apr 6;13:5642. doi: 10.1038/s41598-023-32450-6 (PMC10079669; doi:10.1038/s41598-023-32450-6)

Supplementary information

Floral scent of the Mediterranean fig tree: significant inter-varietal difference but strong conservation of the signal responsible for pollinator attraction

Li Cao, Younes Hmimsa, Salama El fatehi, Bruno Buatois, Marie-Pierre Dubois, Maïlys Le Moigne, Martine Hossaert-McKey, Yildiz Aumeeruddy-Thomas, Anne-Geneviève Bagnères, Magali Proffit

Figure S1. Mean relative proportions of all volatile organic compounds (VOCs) from the different chemical classes emitted by the eight varieties of *Ficus carica* (a) and pairwise comparisons (b). Pairwise comparisons of the relative proportions were conducted using permutational multivariate analysis of variance (PERMANOVA). Significant codes: P < 0.01 “**”, P < 0.05 “*”, P > 0.05 “NS”.

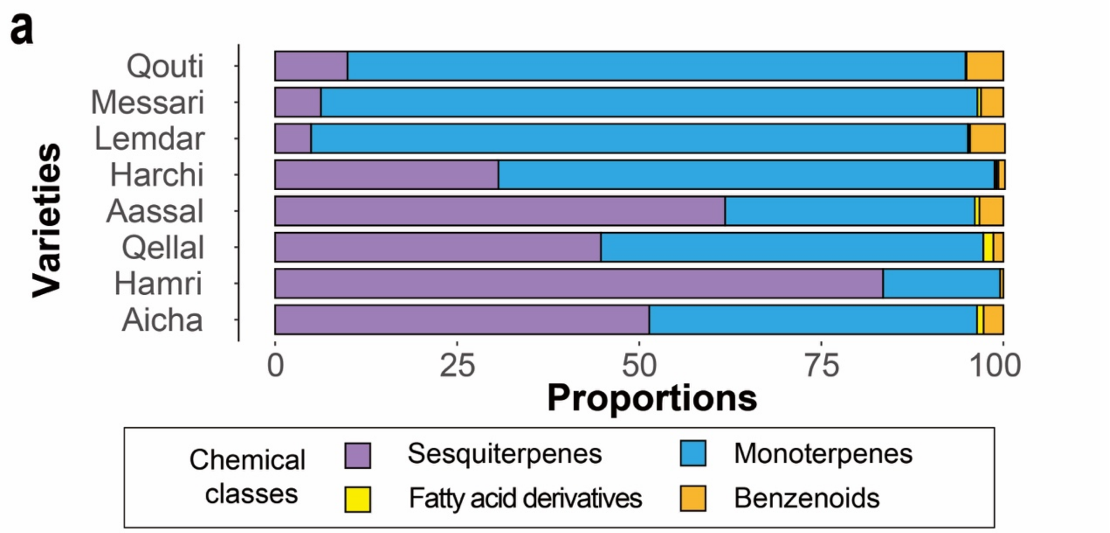

b

	Qouti	Messari	Lemdar	Harchi	Aassal	Qellal	Hamri
Messari	NS						
Lemdar	NS	NS					
Harchi	*	*	**				
Aassal	**	**	**	*			
Qellal	**	*	**	NS	NS		
Hamri	**	**	**	**	NS	**	
Aicha	*	**	**	NS	NS	NS	*

Figure S2. Mean relative proportions of the four attractive volatile organic compounds emitted by the eight varieties of *Ficus carica*.

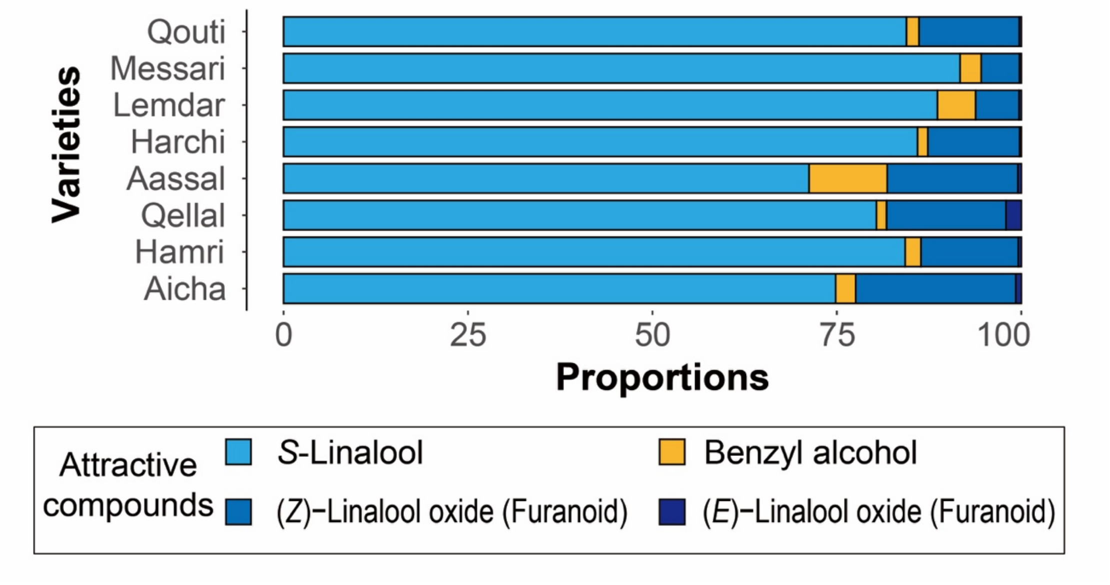

Table S1. Information on the 15 microsatellite markers used for the genetic analysis. Locus name, fluorescent dye-labeled (HEX, FAM and NED fluorochromes), size range of amplified fragments, hybridization temperature, number of individuals analyzed (N_I). Genetic parameters include number of alleles (A) and the genotype number (N_G) per locus.

Locus	Fluorescent dye	Range Size (pb)	Hybridization temperature	N_I	A	N_G
LMFC30	HEX	231-261	50	80	6	8
LMFC32	FAM	197-223	55	80	5	6
FSYC04	HEX	177-193	55	80	3	3
MFC2	HEX	152-190	50	80	4	3
FSYC01	FAM	117-160	50	80	7	7
MFC11	NED	156-203	55	80	2	3
MFC3	FAM	96-144	55	80	4	5
MFC1	HEX	161-199	58	80	3	5
LMFC28	NED	192-202	55	80	4	5
LMFC24	FAM	272-276	55	80	3	4
MFC8	HEX	143-193	50	80	2	2
LMFC26	NED	224-236	55	80	2	2
MFC9	FAM	184-225	58	80	4	4
MFC4	NED	210-226	50	80	2	3
LMFC19	HEX	296-332	50	80	2	2

Figure S3. UPGMA tree showing the relationships among the ten individual trees of each of the eight studied varieties of *Ficus carica*. Nei distance was calculated on 15 SSR markers.

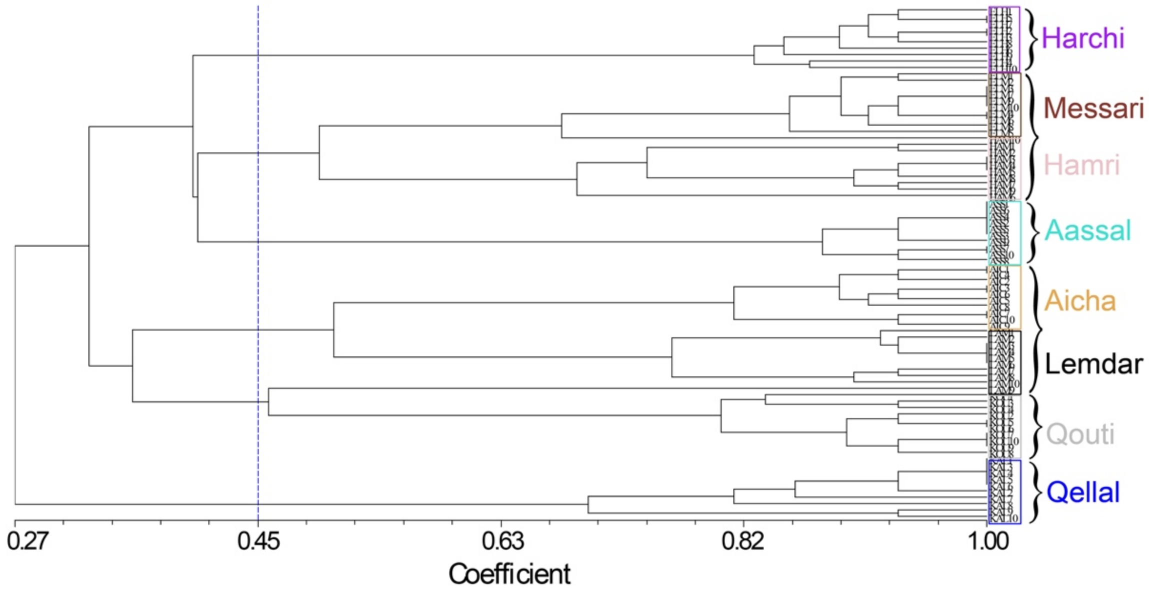

Figure S4. Non-metric multidimensional scaling ordination (NMDS) of the relative proportions of all volatile organic compounds (VOCs) at the three times within the manual pollination period for each of the eight varieties of *Ficus carica* (a, b, c, d, e, f, g and h), based on Bray-Curtis distances. Stress: A = 0.097, B = 0.066, C = 0.105, D = 0.089, E = 0.039, F = 0.085, G = 0.057, H = 0.079. Results of pairwise comparisons between times were performed using permutational multivariate analysis of variance (PERMANOVA) with correction of *p*-values using the fdr (false discovery rate), different letters indicating statistically significant differences.

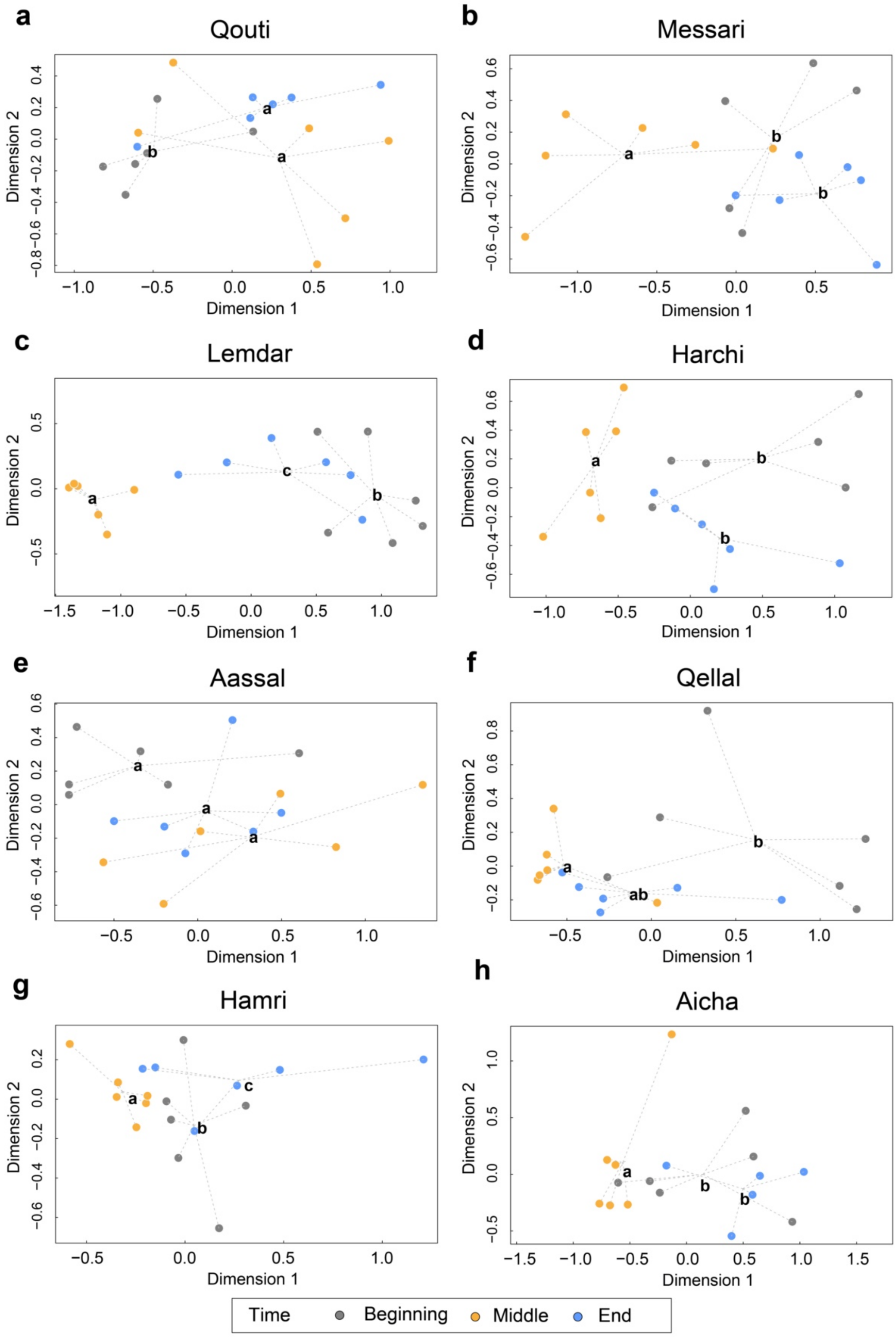

a
Qouti
b
Messari
c
Lemdar
d
Harchi
e
Aassal
f
Qellal
g
Hamri
h
Aicha
Dimension 1
Dimension 2
Time
Beginning
Middle
End

Table S2. Results on comparison among times within the manual pollination period of relative proportions of the four chemical classes of volatile organic compounds (VOCs) for each variety of *Ficus carica* during the manual pollination period. Permutational multivariate analysis of variance (PERMANOVA) was performed on relative proportion of the four chemical classes (standardization before the analysis). Degrees of freedom (Df) and results of the test (F) are indicated. Significant *p*-values ($p < 0.05$) are indicated in bold.

Varieties	Df	all VOCs	
		F	*p*
Qouti	2, 17	3.43	**0.04**
Messari	2, 16	4.73	**0.01**
Lemdar	2, 17	29.28	**< 0.001**
Harchi	2, 17	5.71	**0.01**
Aassale	2, 17	0.98	**0.40**
Qellal	2, 17	8.17	**< 0.001**
Hamri	2, 17	6.27	**< 0.001**
Aicha	2, 16	8.57	**< 0.001**

Figure S5. Mean relative proportions of all volatile organic compounds (VOCs) from the different chemical classes at the three different times within the manual pollination period of the eight varieties of *Ficus carica* (a, b, c, d, e, f, g and h). Results of pairwise comparisons between times were performed using permutational multivariate analysis of variance (PERMANOVA) with correction of *p*-values using the fdr (false discovery rate) procedure. Different letters indicate statistically significant differences.

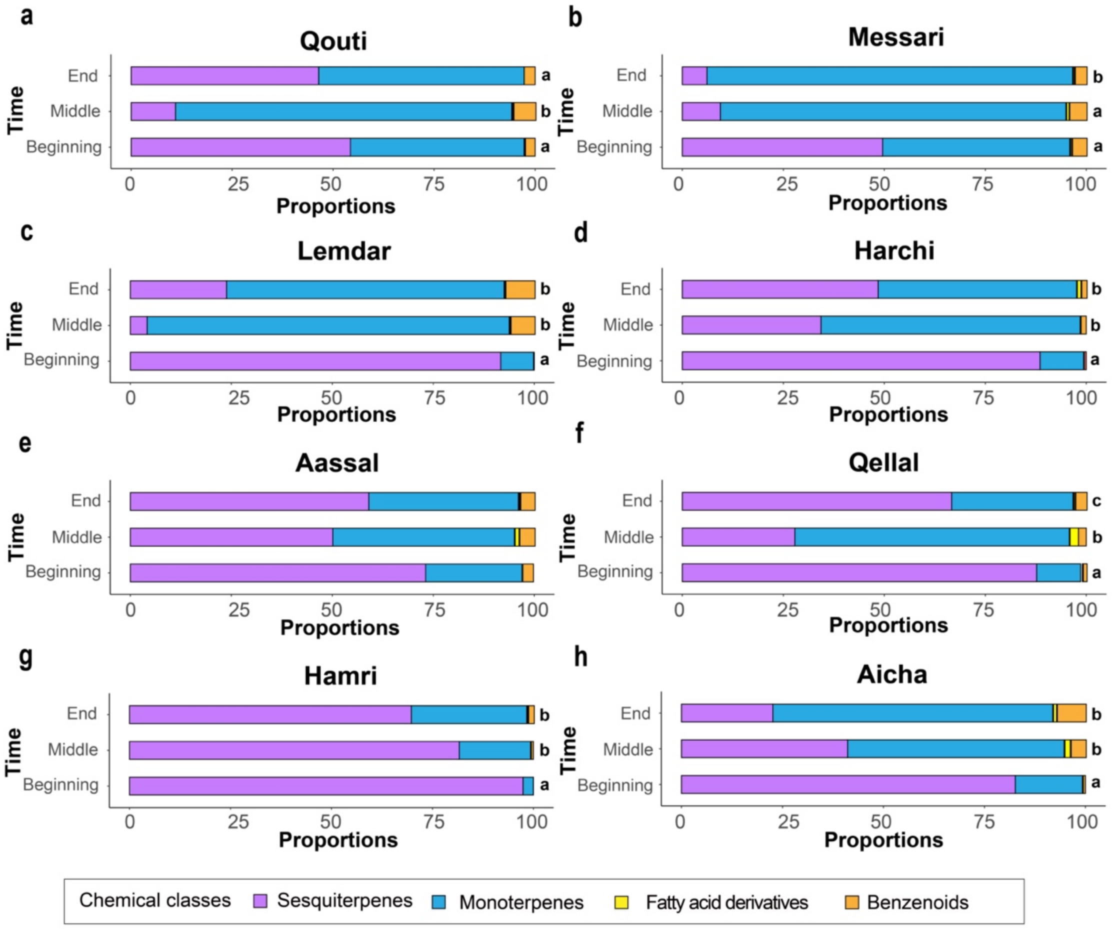

Supplement: Supplementary file 1 — Supplementary Information. [file 41598_2023_32450_MOESM1_ESM.pdf]
